# Microencapsulation of *Lactobacillus plantarum* and *Bacillus subtilis* using baker’s yeast cell wall: characterization and stability assessment under stress conditions

**DOI:** 10.3389/fmicb.2026.1719665

**Published:** 2026-04-01

**Authors:** Saima Rehman, Adnan Hussain Gora, Qurat ul Ain, Shahina Muhammed, Brijit Xavier K., N. K. Sanil, Krupesha Sharma S. R., Kajal Chakraborty

**Affiliations:** 1 Marine Biotechnology, Fish Nutrition and Health Division, ICAR-Central Marine Fisheries Research Institute, Kochi, India; 2 Department of Biotechnology, Cochin University of Science and Technology, Kochi, India

**Keywords:** acid exposure, enzyme exposure, *in vitro*, microencapsulation, probiotics

## Abstract

Yeast cell wall components, being natural, biodegradable, and generally recognized as safe, offer a promising alternative to synthetic encapsulants for probiotic delivery. This study aimed to evaluate baker’s yeast (*Saccharomyces cerevisiae*) cell wall as an encapsulant for improving the stability and gastrointestinal survivability of probiotics. Two probiotic strains with complementary functional traits were selected: *Lactobacillus plantarum* (a non-spore-forming lactic acid bacterium sensitive to gastric stress) and *Bacillus subtilis* (a spore-forming, robust probiotic widely used in feed and pharmaceutical applications). Probiotic cells (≈10^8^–10^9^ colony forming unit mL^–1^) were encapsulated within hollow yeast cell wall particles obtained via sequential acid-alkali treatment. Encapsulation efficiency, particle size, surface charge, structural integrity, and probiotic survival under simulated gastrointestinal conditions were evaluated. Scanning electron microscopy revealed a porous, honeycomb-like yeast cell wall structure (3–6 μm) facilitating probiotic encapsulation. FTIR analysis confirmed the successful encapsulation of *Bacillus subtilis* and *Lactobacillus plantarum* within the yeast cell wall matrix. Spectral changes indicated that encapsulation was driven primarily by non-covalent interactions, dominated by hydrogen bonding between yeast β-glucan hydroxyl groups and probiotic surface biomolecules. Dynamic light scattering showed a narrow and uniform size distribution of unloaded yeast cell wall (D50 = 0.63 μm; span = 0.42), while microencapsulation increased particle size, yielding a relatively uniform distributions for *B. subtilis* (D50 = 0.89 μm; span = 0.79) and a moderately polydisperse profile for *L. plantarum* (D50 = 1.67 μm, span = 1.28). Zeta potential values shifted from −16.4 ± 0.53 mV (unloaded yeast cell wall) to −32.73 ± 1.39 mV (*B. subtilis*) and −30.36 ± 0.42 mV (*L. plantarum*), indicating enhanced colloidal stability (*p* < 0.05). Encapsulation efficiencies were 89.6% ± 3.19% (*B. subtilis*) and 86.57% ± 1.50% (*L. plantarum*), significantly higher than their non-encapsulated counterparts (75.0% ± 2.26% and 40.6% ± 16.3%, respectively; *p* < 0.05). Encapsulated probiotics exhibited significantly improved survival in simulated gastric and intestinal fluids compared with free cells (*p* < 0.05). Baker’s yeast cell wall-based encapsulation significantly enhances probiotic stability, colloidal behavior, and gastrointestinal tolerance through strain-specific physicochemical interactions. This approach offers a safe and effective delivery platform for functional feed and pharmaceutical applications.

## Graphical Abstract

**Figure:**
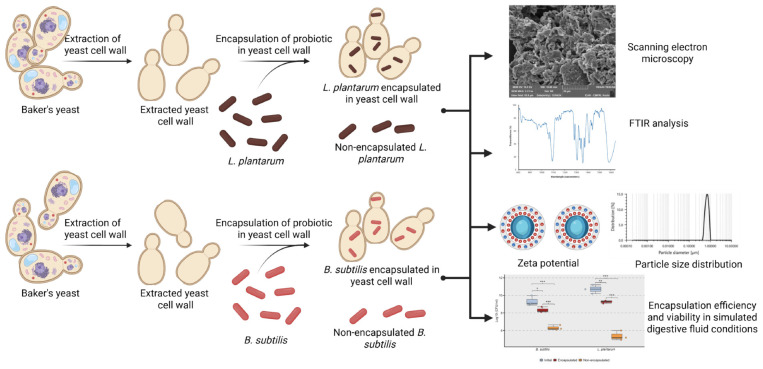
Flowchart depicting encapsulation of *L. plantarum* and *B. subtilis* probiotics using extracted baker’s yeast cell wall followed by characterization through scanning electron microscopy, FTIR analysis, zeta potential, particle size distribution, and evaluations of encapsulation efficiency and probiotic viability in simulated digestive conditions. This figure was created with biorender.com.

## 1 Introduction

Aquaculture represents an important sector contributing substantially to animal protein production. Over the last three decades, the global aquatic animal farming sector has expanded at an average growth rate of 6.7% (Food and Agriculture Organization of the United Nations [FAO], 2022, 2024). This rapid expansion has largely been driven by the intensification of culture systems to meet rising demand. However, intensive management practices have introduced new challenges, such as disease outbreaks, heightened stress and compromised immune function in cultured species (Rehman et al., 2017, 2022a). As a result, antibiotics have been extensively used to control bacterial disease outbreaks, but their indiscriminate application has accelerated the emergence of antimicrobial and multidrug-resistant pathogens (Sharma et al., 2024). These concerns underscore the urgent need to identify sustainable and effective alternatives to antibiotics in aquaculture (Ghosh et al., 2019). Although probiotics are considered promising substitutes for antibiotics, they must reach and establish themselves at the target site of the host’s mucosal tissue at concentrations of at least 10^6^–10^7^ CFU/g to ensure successful colonization (da Silva et al., 2021). Accordingly, food safety authorities recommend that functional feeds fortified with probiotics contain between 10^8^ and 10^9^ CFU/g (Terpou et al., 2019; O’Connell Motherway et al., 2024). However, there are several challenges in achieving and maintaining these recommended levels. Various factors such as conditions during manufacturing, storage, and stressors like acidic gastric pH, concentrated bile salts, and the host’s digestive enzymes can significantly affect the survival of probiotics in the mucosal tissues (Bustos et al., 2025).

Microencapsulation, a widely used immobilization technique, involves entrapping probiotic cells within a protective matrix to enhance their viability and ensure targeted delivery in adequate amounts to the host (Sbehat et al., 2022). Among the various encapsulating materials, yeast cells have recently attracted interest as viable carriers (Liu et al., 2016). Yeast cell wall is primarily composed of polysaccharides and glycoproteins (up to 85% and 15%, respectively) as the primary building blocks. The cell wall of *Saccharomyces cerevisiae* or baker’s yeast is 100–200 nm thick, with β-glucans as the primary polysaccharide, covering 50%–60% of the cell wall mass. Depending on the probiotic to be encapsulated and the encapsulation method used, intact yeast cells, yeast cell walls, or yeast-derived glucan particles may be selected for encapsulation (Günal-Köroğlu et al., 2024). However, the restricted permeability of intact yeast cells limits their application as protective agents or delivery vehicles for probiotics. To overcome this limitation, pretreatment strategies are employed to increase the porosity and permeability of yeast cell structures, improving the encapsulation efficiency (Dadkhodazade et al., 2021; Tan et al., 2021). For instance, crushed *S. cerevisiae* cell wall effectively coated alginate-encapsulated *Lactobacillus acidophilus* and *Bifidobacterium bifidum*, enhancing their survival under simulated gastrointestinal conditions (Mokhtari et al., 2017). Similarly, yeast β-glucan used as a protective agent against freeze-dry damage enhanced the cryoprotection, adhesion, and exopolysaccharide production of the probiotic *L. plantarum* 201 (da Silva Guedes et al., 2019). A 3% yeast cell cross-linked with β-glucan wall showed the highest encapsulation efficiency (97.6%), superior survival in simulated gastrointestinal fluid (8.77 log CFU/mL vs. 4 log CFU/mL for free cells), and maintained the viability of spray-dried *L. acidophilus* LA-5 (above 6 log units after 56 days), demonstrating a predicted shelf life of 200 days at 4 °C (Ahmad et al., 2019). However, there are few studies that have specifically evaluated the cell wall of baker’s yeast as an encapsulating material for probiotics *L. plantarum* and *Bacillus subtilis*.

*L. plantarum* and *B. subtilis* represent two functionally distinct yet industrially relevant probiotics. *L. plantarum*, a non-spore-forming lactic acid bacterium widely recognized for its immunomodulatory capacity, mucosal adhesion ability, antimicrobial metabolite production, and proven benefits in gut health; however, its viability is often compromised under gastric acidity, bile stress, and feed processing conditions (Fidanza et al., 2021). In contrast, *B. subtilis* is a spore-forming probiotic characterized by exceptional resistance to environmental stress, long shelf life, and suitability for large-scale feed applications, with additional benefits including enzyme production and pathogen inhibition (Stülke et al., 2023). The contrasting physiological traits of these strains provide a robust framework to evaluate whether a yeast cell wall–based matrix can enhance probiotic protection across both stress-sensitive and stress-resistant probiotic types, thereby strengthening the translational relevance of the encapsulation strategy for industrial and aquaculture applications.

Baker’s yeast is a widely used and commercially available product for human consumption all over the globe (Bai et al., 2022). Using commercial food-grade baker’s yeast is crucial for the safety and acceptance of yeast-probiotic formulations. Despite extensive research on probiotic microencapsulation, most existing approaches rely on conventional polymeric matrices or chemically modified yeast derivatives, which increase processing complexity, cost, and limit scalability. This study addresses this gap by utilizing *S. cerevisiae* cell wall as a natural, biofunctional encapsulant, that provide probiotic protection and gastrointestinal survivability. By preserving the multilayered organization of the Baker’s yeast cell wall composed mainly of β-glucans, mannoproteins, and chitin, this approach enables the cell wall to function as a physical protective barrier for probiotic cells. β-glucans and chitin confer rigidity and structural stability to the yeast cell wall and resistance to environmental stressors such as acidic pH, bile salts, and dehydration, whereas mannoproteins, predominantly located in the outer layer, govern surface adhesion, enhancing probiotic retention and stability (Günal-Köroğlu et al., 2024; Ismail et al., 2025). The objective of the present study was to evaluate the efficacy of food-grade *S. cerevisiae* cell wall as a stand-alone, natural microencapsulation system for probiotics *L. plantarum* and *B. subtilis*, focusing on encapsulation efficiency, structural characteristics, and survivability under simulated gastrointestinal conditions to assess its potential as an alternative to conventional encapsulants.

## 2 Materials and methods

### 2.1 Materials

All chemicals and reagents used in this study were of analytical grade unless otherwise stated. De Man, Rogosa and Sharpe (MRS) broth and agar, Luria–Bertani (LB) broth and agar were obtained were obtained from HiMedia Laboratories (Mumbai, India). Luria–Bertani (LB) broth and agar were obtained from HiMedia Laboratories (Mumbai, India). Tween 80 (polyoxyethylene sorbitan monooleate; CAS No. 9005-65-6), glycerol (CAS No. 56-81-5), sodium hydroxide (CAS No. 1310-73-2), and hydrochloric acid (CAS No. 7647-01-0) were procured from Merck (India). Phosphate-buffered saline (PBS, pH 7.4) was prepared according to standard protocols. The probiotic strains *L. plantarum* (MTCC 1325) and *B. subtilis* (MTCC 3055) were procured from the Microbial Type Culture Collection and Gene Bank (MTCC), CSIR–Institute of Microbial Technology, Chandigarh, India. Lyophilized cultures were revived and maintained according to MTCC guidelines. Commercially available Baker’s yeast (*S. cerevisiae*) was procured from a local certified supplier (Prions Biotech, Karnataka, India) and authenticated prior to use. Simulated gastric fluid (SGF) was prepared using pepsin from porcine gastric mucosa (≥250 U mg^–1^ protein; CAS No. 9001-75-6, Sigma-Aldrich, USA) dissolved in sterile saline and adjusted to pH 2.0. Simulated intestinal fluid (SIF) was prepared using pancreatin from porcine pancreas (activity ≥ 4 × USP specifications; CAS No. 8049-47-6, Sigma-Aldrich) supplemented with trypsin from porcine pancreas (≥10,000 BAEE U mg^–1^ protein; CAS No. 9002-07-7, Sigma-Aldrich), and adjusted to pH 7.5. All enzymes were freshly prepared prior to use to ensure consistent enzymatic activity.

### 2.2 Extraction of yeast cell wall for microencapsulation

The yeast cell extraction method was adapted from Lin et al. (2021) with modifications. Briefly, 20 g of baker’s yeast was suspended in 300 mL of 1 M sodium hydroxide, heated at 80 °C for 1.5 h, and centrifuged at 3000 rpm for 10 min. The pellet was washed twice with deionized water, then resuspended in hydrochloric acid (pH 4) and incubated at 60 °C for 1.5 h. After a final centrifugation (3000 rpm, 10 min) and two additional washes with deionized water, the pellet was sequentially rinsed with 100% isopropyl alcohol (four times) and acetone (twice). Following centrifugation, the pellet was air-dried overnight at room temperature. To prevent particle aggregation, the yeast cells were sonicated (Sonics, UK) for 10 min in 100–200 mL of deionized water at 20 kHz, then homogenized (IKA™) at 10,000 rpm for 10 min. The suspension was frozen at −80 °C, lyophilized, and the resulting powder was stored at 4 °C for further analysis.

### 2.3 Bacterial culture and growth curve

The growth curves of *B. subtilis* and *L. plantarum* were performed to corroborate their growing stage and determine the appropriate time (early stationary phase) for the bacteria to be complexed with the yeast microcapsules. *L. plantarum* was inoculated into 150 mL of sterile MRS broth and incubated at 37 °C. At 4- to 8-h intervals, 1 mL aliquots were withdrawn, centrifuged at 3000 rpm for 10 min at room temperature, and washed twice with sterile PBS (pH 7.4). The final cell pellet was resuspended in 1 mL of PBS, and 200 μL of the suspension was transferred to a 96-well microtiter plate. Optical density (OD) was measured at 600 nm using PBS as the blank. The OD curve was further analyzed to determine viable cell counts and correlate them with OD readings. *L. plantarum* cultures grown in MRS broth were subjected to serial dilution and subsequent spread plate analysis. Simultaneously, OD_600_ was recorded for each dilution. Plates were incubated at 37 °C for 24 h, and CFU/ml were calculated. A similar protocol was followed for *B. subtilis* except it was grown in LB broth and LB agar to assess growth and viability.

### 2.4 Encapsulation of bacteria with baker’s yeast cell wall

*L. plantarum* was cultured in 100 mL MRS broth at 37 °C for 24 h under shaking conditions (150 rpm), while *Bacillus subtilis* was cultured in LB broth at 37 °C for 24 h (150 rpm). Both strains were harvested at the late exponential growth phase by centrifugation (3000 rpm, 10 min, room temperature), washed twice with sterile deionized water, and standardized to a cell density of approximately 10^9^–10^9^ CFU mL^–1^ prior to encapsulation. Briefly, 100 mg of yeast cell wall material was dispersed in 10 mL of sterile deionized water containing 0.02% (v/v) Tween-80 and homogenized at 1500 rpm for 10 min to obtain a uniform suspension. The standardized bacterial pellet was then mixed with the yeast cell wall suspension at a defined biomass-to-encapsulant ratio (approximately 1:10, wet biomass to dry yeast cell wall, w/w). The mixture was gently vortexed for 5 min to promote encapsulation of bacterial cells within the porous yeast cell wall matrix, followed by centrifugation at 7000 rpm for 15 min to collect the encapsulated cells. The resulting pellet was washed once with sterile phosphate-buffered saline (PBS; 7000 rpm, 10 min) to remove unbound bacteria. The encapsulated probiotic pellet was resuspended in 5 mL of sterile 5% (v/v) glycerol as a cryoprotectant, poured into sterile petri plates, frozen at −80 °C overnight, and subsequently lyophilized to obtain dry encapsulated probiotic powders. The same encapsulation procedure was applied to both *L. plantarum* and *B. subtilis* (Shah et al., 2016; Gani et al., 2018).

### 2.5 Encapsulation efficiency and viability of probiotics

The encapsulation efficiency of yeast cell wall for *L. plantarum* and *B. subtilis* was calculated following the method of a previous study (Ahmad et al., 2019) with some modifications. To release the bacteria from the microcapsules, 100 mg of microcapsules were dissolved in 2.5% peptone with the aid of a magnetic stirrer. The cell viability was checked by enumeration on LB agar for *B. subtilis* and on MRS agar for *L. plantarum*. The plates were placed in an incubator (TE 392/170L, Tecnal, Piracicaba, SP, Brazil) under anaerobic conditions at 37 °C for 24–48 h. Plates containing 20–300 colonies were counted and recorded as CFU per gram of dried powder or per mL of solution. A parallel group of non-encapsulated bacteria was also processed using the same procedure to evaluate the protective effect of encapsulation by yeast cell wall. The viability in the resulting three groups (initial, final encapsulated and final non-encapsulated) was determined as the fraction of viable cells in microcapsules over the viable cells in the feed solution before the freeze-drying process.


V⁢i⁢a⁢b⁢i⁢l⁢i⁢t⁢y=[l⁢o⁢g⁢(f⁢i⁢n⁢a⁢l⁢C⁢F⁢U/m⁢l)/l⁢o⁢g⁢(i⁢n⁢i⁢t⁢i⁢a⁢l⁢C⁢F⁢U/m⁢l)]×100

log (final CFU/ml) = Colony count of bacteria obtained at the end of the preparative process (encapsulated or non-encapsulated) per ml of formulation.

log (initial CFU/ml) = Colony count of viable bacteria initially included in the preparative process of micro particles.

### 2.6 Scanning electron microscopy

The lyophilized samples were placed on adhesive tape affixed to a circular aluminum specimen stub (Ahmad et al., 2019). After being sputter-coated vertically with gold–palladium, the samples were imaged at an accelerator potential of 10 kV using a scanning electron microscope (Hitachi S-300H, Tokyo, Japan).

### 2.7 Fourier transforms infrared spectroscopy (FTIR)

The particles of lyophilized yeast cell wall and microencapsulated *B. subtilis* and *L. plantarum* with yeast cell wall were made into thin powder and mixed with KBr to make pellets (Ahmadi et al., 2025). These pellets were analyzed using Thermo Nicolet 6700 FTIR spectrophotometer in the infrared spectral range from 500 to 4000 cm^1^ to understand the surface chemistry of each sample.

### 2.8 Zeta potential and particle size determination

Particle size and zeta potential of *B. subtilis* and *L. plantarum* encapsulated in baker’s yeast cell wall were evaluated by dynamic light scattering (DLS) measured using the Litesizer™ 500 (Anton Paar, Virginia, USA). For the analysis, a sample (0.01%, w/v) was suspended in de-ionized water (Elix-10, Millipore, Molsheim, France) and sonicated to fully disperse the particles (Ashraf et al., 2021). The span value, which is a dimensionless number indicating the width of the particle size distribution was calculated using the formula:


$$S\,p\,a\,n=D_{90}-D_{10}/D_{50}$$

Where D_10_ is the diameter below which 10% of the particles fall, D_50_ is the median particle diameter (50% of particles fall) and D_90_ is the diameter below which 90% of the particles fall. Lower span values represent a narrower and more uniform particle size distribution, while higher values indicate greater polydispersity. Hydrodynamic diameter reflects the effective particle size in solution, accounting for the particle core and its associated solvation layer.

### 2.9 Viability test in simulated gastric fluid

Simulated gastric fluid (SGF) was prepared by dissolving 2 g of sodium chloride (NaCl) in approximately 800 mL of deionized water, followed by the addition of 2 mL of concentrated hydrochloric acid (HCl). The volume was then made up to 1 L with deionized water and mixed thoroughly to obtain the SGF stock solution (Shah et al., 2016). For enzymatic simulation, 0.064 g of pepsin was dissolved in 20 mL of the SGF stock, and the pH of the resulting solution was adjusted to 2.5 using dilute HCl or sodium hydroxide (NaOH) as necessary. A known amount of encapsulated and non-encapsulated *L. plantarum* and *B. subtilis* (lyophilized) were weighed and suspended in 200 μL of the freshly prepared SGF solution. The samples were incubated at 37 °C with agitation at 100 rpm. Aliquots were collected at 0 min, 30 min and 60 min. At each time point, samples were subjected to serial dilution in sterile PBS. From each dilution, 100 μL was plated onto sterile MRS or LB agar plates. Plates were incubated at 37 °C for 24 h. After incubation, CFU were counted to determine the number of viable bacteria at each time interval.

### 2.10 Viability test in simulated intestinal fluid

Simulated intestinal fluid (SIF) was prepared by dissolving 10 g of trypsin, 10 g of pancreatin, 3 g of bile salts, and 8.5 g of sodium chloride in 1 L of deionized water and adjusted to pH 7.4 (Ahmadi et al., 2025). The solution was mixed thoroughly until all components were completely dissolved and was used fresh or stored appropriately for further experimental applications. Viability of encapsulated and non-encapsulated bacteria was checked as mentioned in Section 2.9.

### 2.11 Statistical analysis

The statistical analysis was performed in R version 4.3.1. The data were checked for normality and homoscedasticity by Shapiro-Wilk and Bartlett’s test, respectively. Parametric one-way ANOVA was performed where the assumptions of ANOVA were met. In the case of non-parametric data, statistical differences were identified using the Kruskal-Wallis test. Duncan’s multiple range test (confidence level = 0.05) and Dunn’s test were employed to understand the statistical differences between treatments in the case of parametric and non-parametric datasets, respectively. All experiments were conducted in triplicates. Results are expressed as mean ± standard error, with a significance level set at *p* < 0.05. The R Package ggplot2 was used for data visualization (Wickham, 2016).

## 3 Results

### 3.1 SEM analysis

**FIGURE 1:**
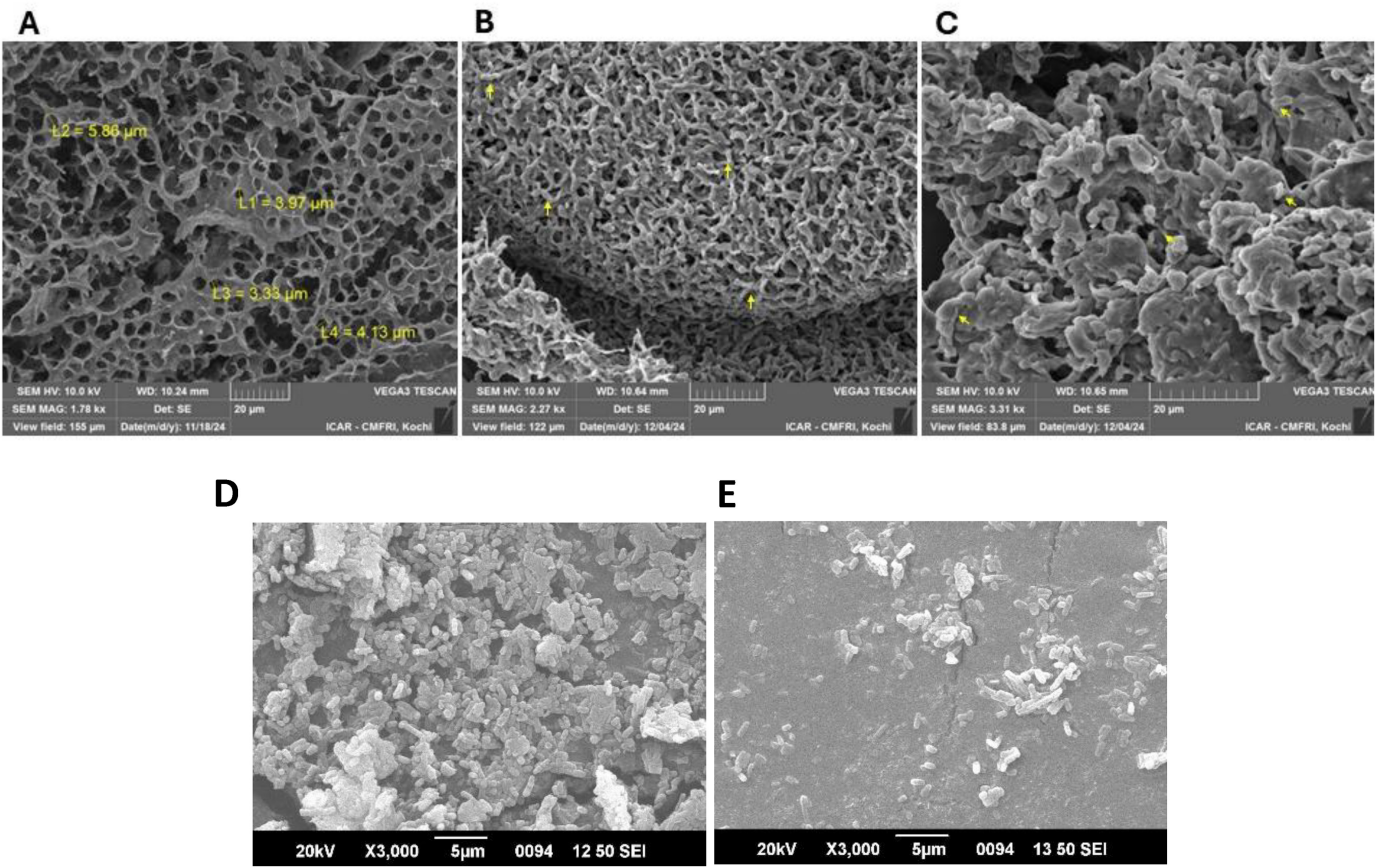
Scanning electron micrograph of the extract yeast cell wall before and after the probiotic encapsulation. **(A)** Representative electron microscopy image shows mesh-like architecture of yeast cell wall having a pore size of 3–6 μm. **(B)** The probiotic *B. subtilis* loaded into the empty yeast cell wall. **(C)** The microencapsulation of *L. plantarum* causes a slight aggregative effect on the yeast cell wall. **(D,E)** Electron microscopy images of *B. subtilis* and *L. plantarum*, respectively.

The surface morphology of the freeze-dried microcapsules of yeast cell wall revealed an intact, porous structure with a honeycomb-like appearance (Figure 1A). The average pore size ranged from 3 to 6 μm, indicating a highly reticulated surface that is suitable for microbial attachment. The SEM micrographs of freeze-dried encapsulated yeast cell wall containing *Bacillus subtilis* revealed a structure with bacterial cells distributed randomly in the matrix (Figure 1B). In case of *L. plantarum*, microencapsulation, the bacterial cells appeared attached, and the interaction of the probiotic with the cell wall resulted in clumping and aggregation of the cells (Figure 1C). The individual SEM images of *B. subtilis* and *L. plantarum* are shown in Figures 1D, E, respectively.

### 3.2 FTIR analysis

**FIGURE 2:**
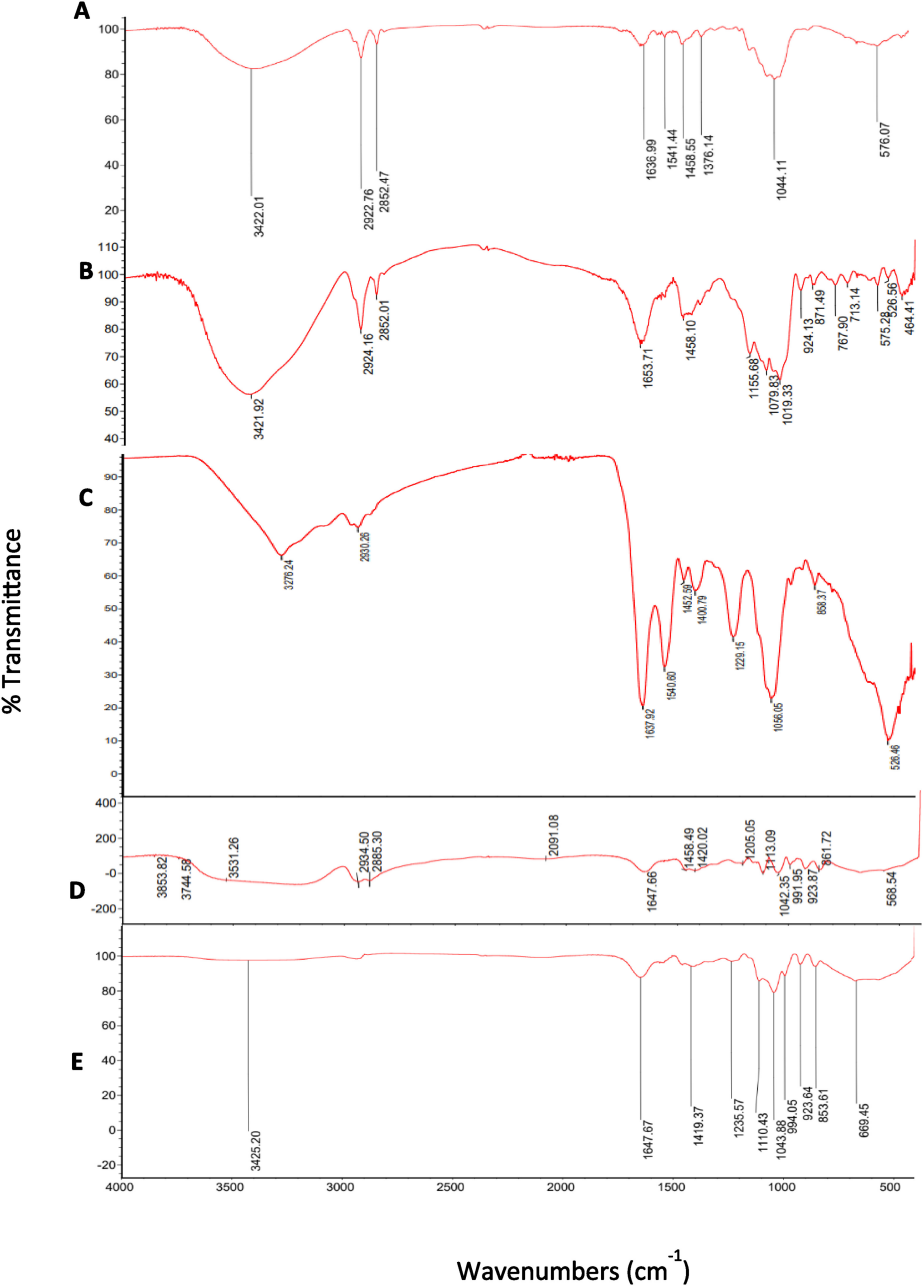
Fourier transform infrared (FTIR) spectra of yeast cell wall before and after the probiotic encapsulation **(A)** native yeast cell wall of *S. cerevisiae* **(B,C)** FTIR of the probiotic *B. subtilis* and *L. plantarum*, respectively. **(D)** *B. subtilis* encapsulated in yeast cell wall **(E)** *L. plantarum* encapsulated in yeast cell wall.

The FTIR spectrum of yeast cell wall (Figure 2A) revealed key functional groups: O–H stretching (3422.01 cm^–1^), C–H stretching (2922.76, 2852.47 cm^–1^), H–O–H bending (1636.99 cm^–1^), CH_2_ deformation (1458.55 cm^–1^), and C–H bending (1376.14 cm^–1^). Polysaccharide-specific features included glycosidic C–O–C and C–O stretching (1044.11 cm^–1^). The FTIR spectrum of *B. subtilis* (Figure 2B) exhibited characteristic peaks for proteins (amide I at 1653.71 cm^–1^), lipids (C–H stretching at 2924.16 and 2852.01 cm^–1^), and polysaccharides (C–O–C stretching at 1155.68 cm^–1^, C–O stretching at 1079.83 and 1019.33 cm^–1^). Structural carbohydrate vibrations were noted at 934.13 and 871.49 cm^–1^, while peaks at 575.28, 526.56, and 464.41 cm^–1^ indicated phosphate or skeletal features. The FTIR spectrum of *Lactobacillus plantarum* (Figure 2C) showed a broad O–H/N–H stretching band at 3276 cm^–1^ and aliphatic C–H stretching at 2930 cm^–1^, indicating the presence of polysaccharides and membrane lipids. Prominent protein-associated bands were observed at 1637 cm^–1^ (amide I) and 1540 cm^–1^ (amide II). Peaks at 1452 and 1401 cm^–1^ corresponded to CH_2_/CH_3_ deformation vibrations. Strong absorptions in the fingerprint region, particularly at 1229 and 1056 cm^–1^, were attributed to phosphate groups and C–O/C–O–C stretching of cell wall polysaccharides, respectively. The FTIR spectrum of *B. subtilis* encapsulated within yeast cell wall (Figure 2D) revealed significant peaks for hydroxyl groups (3853.82, 3744.58, and 3531.26 cm^–1^), CH_2_ stretching (2934.50 and 2885.30 cm^–1^), and protein-related amide I bands (1647.66 cm^–1^). Polysaccharide-specific peaks included glycosidic (1205.05, 1113.09 cm^–1^) and C–O (1042.35 cm^–1^) stretching vibrations, while carbohydrate structures were confirmed by peaks at 991, 923.87, and 861.72 cm^–1^. Furthermore, additional absorptions peaks in the FT-IR spectra between 1200 and 900 cm^–1^ around 1400 cm^–1^ correspond to the presence of bacteria within the yeast matrix. The FTIR spectrum of yeast cell wall–encapsulated *L. plantarum* (Figure 2E) exhibited a broad O–H/N–H stretching band around ∼3400 cm^–1^, similar to the unencapsulated bacteria but with reduced intensity and slight broadening. Compared to free *L. plantarum*, the characteristic amide I and amide II bands (∼1637 and ∼1540 cm^–1^) showed attenuation and partial overlap in the encapsulated spectrum. In contrast, carbohydrate-associated bands in the 1200–900 cm^–1^ region became more prominent following encapsulation, corresponding to C–O and C–O–C stretching.

### 3.3 Particle size and zeta potential

**FIGURE 3:**
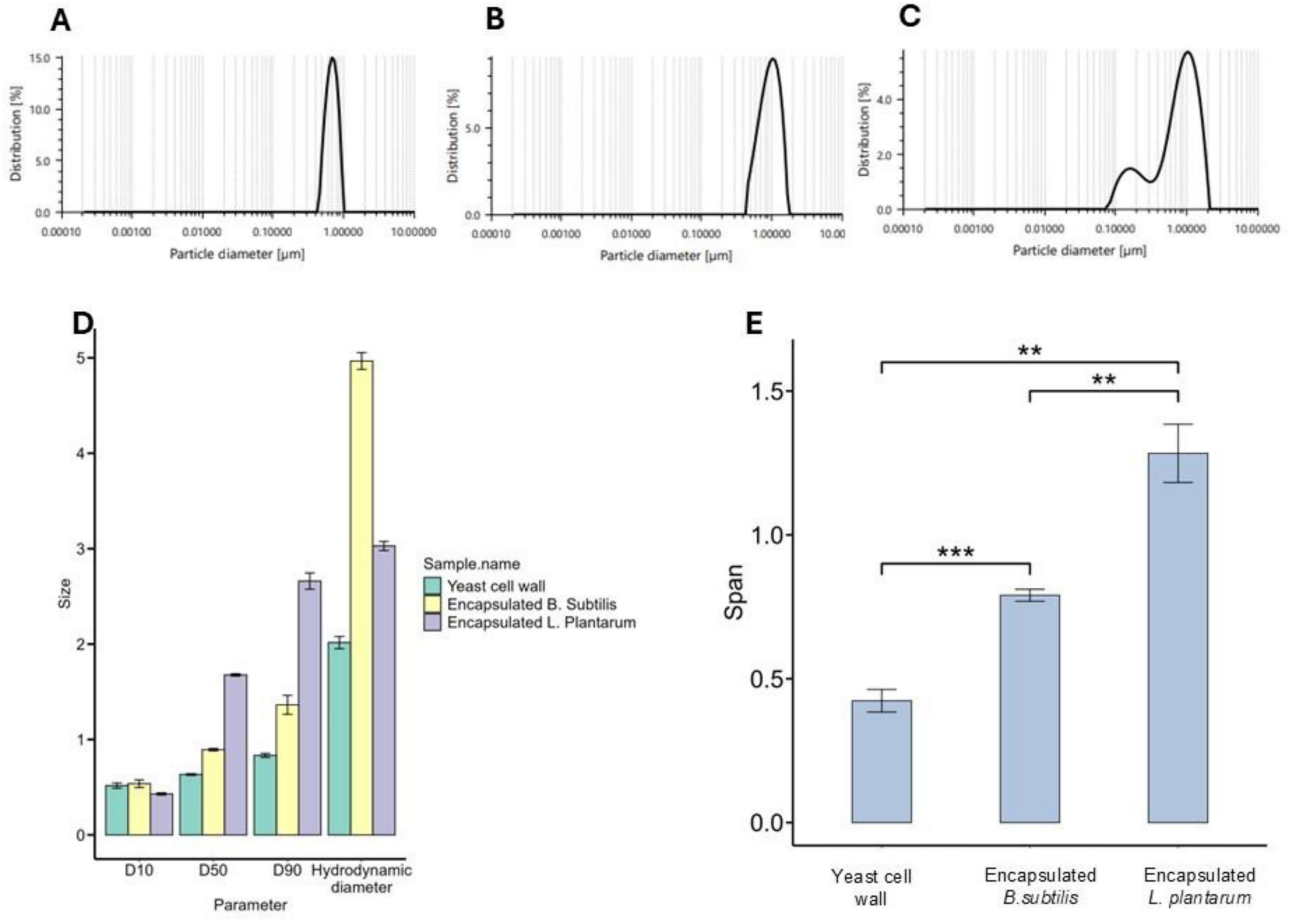
Particle size distribution and hydrodynamic characteristics of yeast cell wall before and after probiotic encapsulation. **(A)** Particle size distribution of *Saccharomyces cerevisiae* cell wall showing a narrow, uniform profile. **(B)** *Bacillus subtilis* encapsulated in yeast cell wall. **(C)** *Lactobacillus* plantarum encapsulated in yeast cell wall. **(D)** Bar plot showing particle size parameters (D10, D50, D90) and hydrodynamic diameter of native yeast cell wall and probiotic-encapsulated formulations. **(E)** Bar plot showing span values of native and encapsulated formulations. Statistical significance: **p < 0.01, ***p < 0.001.

The particle size distribution profile of the yeast cell wall exhibited D50 of 0.63 ± 0.008, a D90 of 0.83 ± 0.02, a hydrodynamic diameter of 2.01 ± 0.06 μm and a calculated span of 0.42 ± 0.03, indicating a relatively narrow and uniform size distribution. In contrast, the microencapsulation of *B. subtilis* within the yeast cell wall matrix showed D50 of 0.89 ± 0.01, a D90 of 1.36 ± 0.09, a hydrodynamic diameter of 4.9 ± 0.08 μm and a calculated span value of 0.79 ± 0.02 reflecting a relatively uniform distribution. Furthermore, the encapsulation of *L. plantarum* showed a D50 of 1.67 ± 0.01, a D90 of 2.66 ± 0.08, a hydrodynamic diameter of 3.02 ± 0.02 μm and a calculated span value of 1.28 ± 0.10 suggesting a moderately polydisperse distribution (Figures 3A–E). Span is calculated using the formula (D90–D10)/D50, where lower values represent a narrower and more uniform particle size distribution, while higher values indicate greater polydispersity.

**FIGURE 4:**
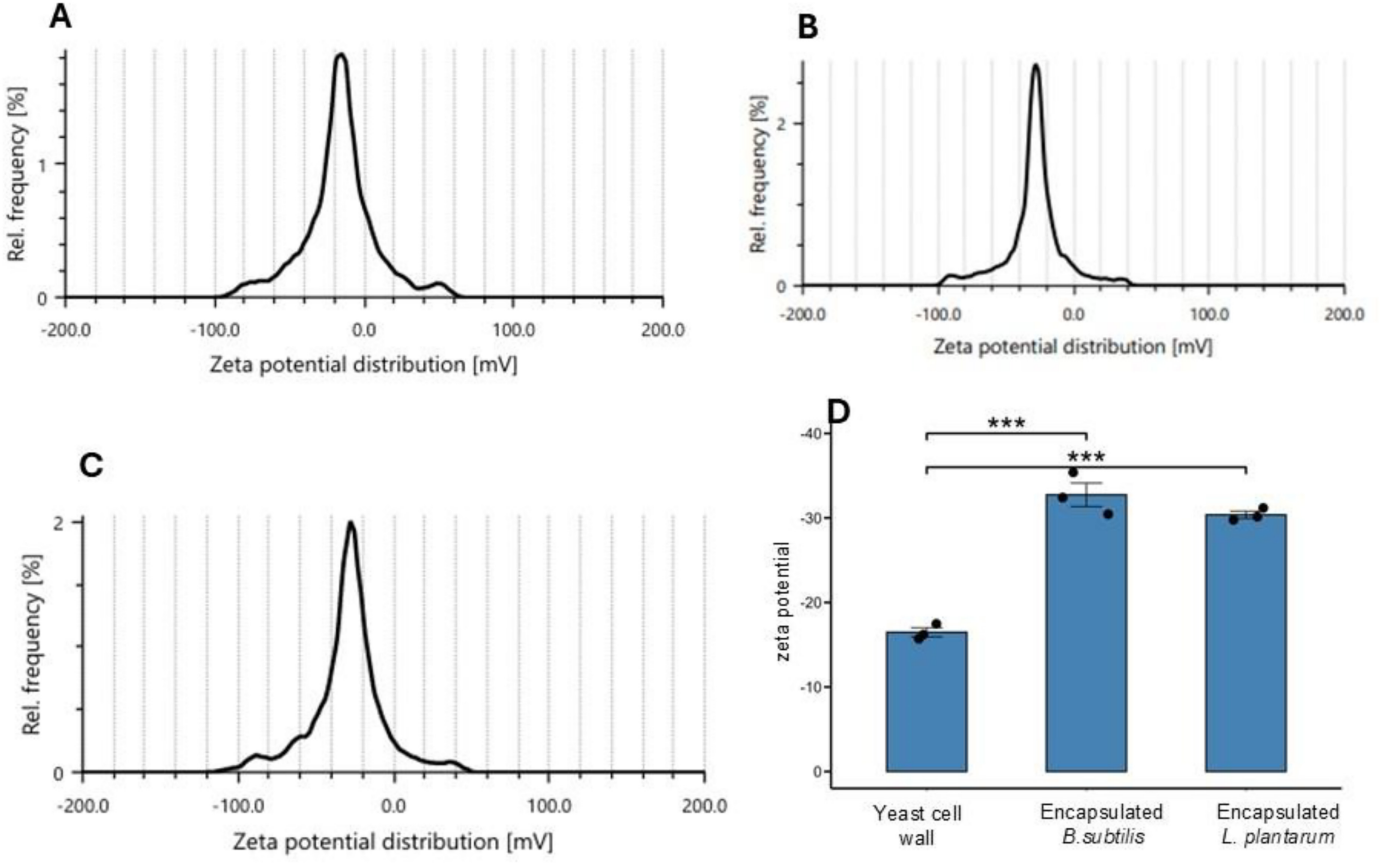
Zeta potential distribution of the yeast cell wall before and after the probiotic encapsulation. **(A)** Native yeast cell wall of *S. cerevisiae* **(B)** *B. subtilis* encapsulated in yeast cell wall **(C)** *L. plantarum* encapsulated in yeast cell wall. **(D)** Bar plot of the zeta potential analysis of the yeast cell wall, encapsulated *B. subtilis* and *L. plantarum* (***p < 0.001).

The zeta potential analysis of the yeast microcapsules revealed a mean surface charge of −16.4 ± 0.53 mV (Figure 4A) indicating a moderately stable colloidal system and sufficient electrostatic repulsion among particles to prevent aggregation. On the other hand, the zeta potential analysis of yeast cell wall encapsulated *B. subtilis* (Figure 4B) and *L. plantarum* (Figures 4C,D) revealed mean surface charges of −32.73 ± 1.39 and −30.36 ± 0.42 mV, respectively, with a strong negative potential suggesting enhanced colloidal stability due to effective electrostatic repulsion among particles.

### 3.4 Bacterial growth curve analysis

**FIGURE 5:**
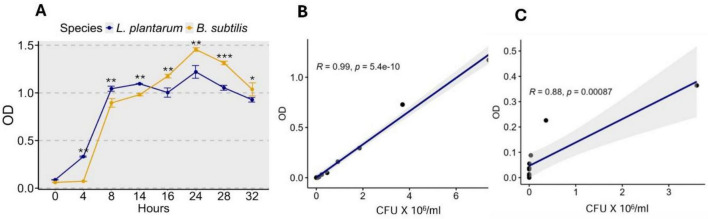
Growth patterns and correlation between optical density and colony-forming units (CFU) for *L. plantarum* and *B. subtilis*. **(A)** The figure illustrates the growth kinetics of *L. plantarum* and *B. subtilis* in culture, monitored by measuring optical density at 600 nm (OD_600_) over time. Concurrently, viable cell counts were determined for *L. plantarum* **(B)** and *B. subtilis* **(C)** at selected time points using serial dilution and plate counting to estimate CFU/mL. (***p < 0.001, **p < 0.01, and *p < 0.05).

The growth profile of *L. plantarum* and *B. subtilis*, monitored over 32 h via optical density (OD), exhibited distinct patterns (Figure 5A). Both strains showed a typical sigmoidal growth trend, with lag phases extending up to 4 h that was particularly pronounced in *B. subtilis*. *L. plantarum* reached its maximum OD of 1.22 ± 0.11 at 24 h, while *B. subtilis* exhibited a more pronounced growth, peaking at an OD of 1.45 ± 0.03 OD at the same time point. After 24 h, both species showed a gradual decline in OD, indicating entry into the stationary or early decline phase. The higher OD of *B. subtilis* throughout the logarithmic and stationary phases suggests a more robust proliferation compared to *L. plantarum* under the tested conditions. Standard curves showed a strong positive linear relationship between OD and CFU for both bacterial strains, with a statistically significant correlation coefficient of R^2^ = 0.99 and 0.88 for *L. plantarum* and *B. subtilis*, respectively (Figures 5B,C). These curves were used to estimate bacterial concentration in experimental preparations based on OD measurements, enabling accurate dosing in subsequent assays.

### 3.5 Viability of *Bacillus subtilis* and *Lactobacillus plantarum* after encapsulation

**FIGURE 6:**
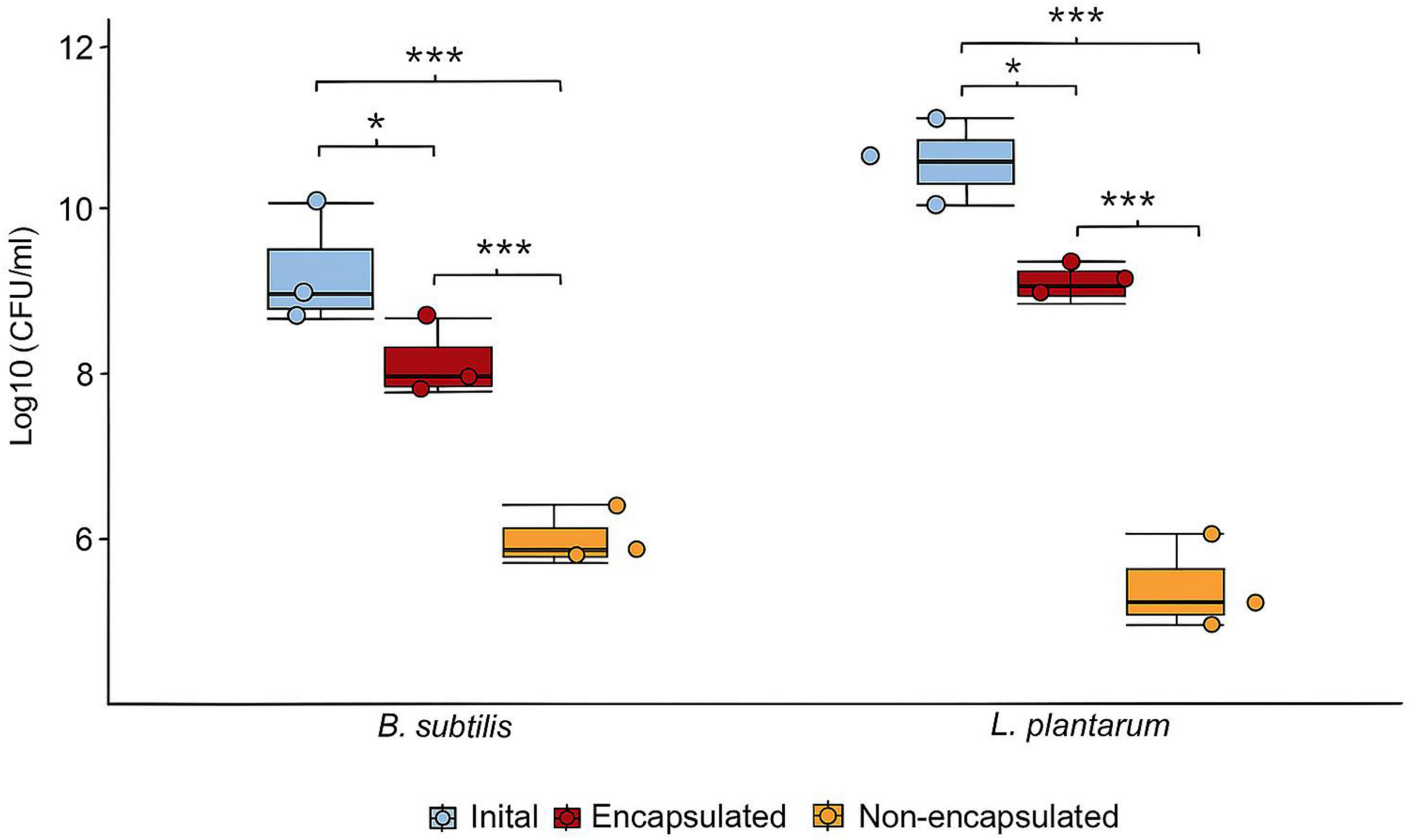
Encapsulation efficiency of *L. plantarum* and *B. subtilis* in yeast cell wall particles based on microbial viability *in vitro*. The figure presents the initial bacterial load (CFU/mL) prior to encapsulation (blue boxplot), along with the final counts of encapsulated (red) and non-encapsulated (tan) cells after the encapsulation process for both *L. plantarum* and *B. subtilis* (*n* = 3, ***p < 0.001, and *p < 0.05).

The viability of *B. subtilis* and *L. plantarum* was evaluated before and after encapsulation by measuring CFU/ml (Figure 6). Both strains had a high initial bacterial load of approximately Log_10_ CFU/ml values of 9.33 ± 0.27 and 10.70 ± 0.23 for *B. subtilis* and *L. plantarum*, respectively. After encapsulation, a significant reduction in CFU was observed in both strains. For *B. subtilis*, the count decreased to 8.34 ± 0.14 Log_10_ CFU/ml in the encapsulated group (89.6% ± 3.19% viability), while the non-encapsulated group dropped more sharply to 6.31 ± 0.13 Log_10_ CFU/ml with 75% ± 2.26 % viability (*p* < 0.001). Similarly, *L. plantarum* showed significantly (*p* < 0.001) better survival in the encapsulated form 86.57% ± 1.5 % viability with viable count of 9.25 ± 0.07 Log_10_ CFU/ml compared to the non-encapsulated group that showed a count of 3.76 ± 1.51 Log_10_ CFU/ml and 40.6% ± 16.3% viability.

### 3.6 Viability of *Bacillus subtilis* and *Lactobacillus plantarum* after encapsulation in simulated gastric fluid

**FIGURE 7:**
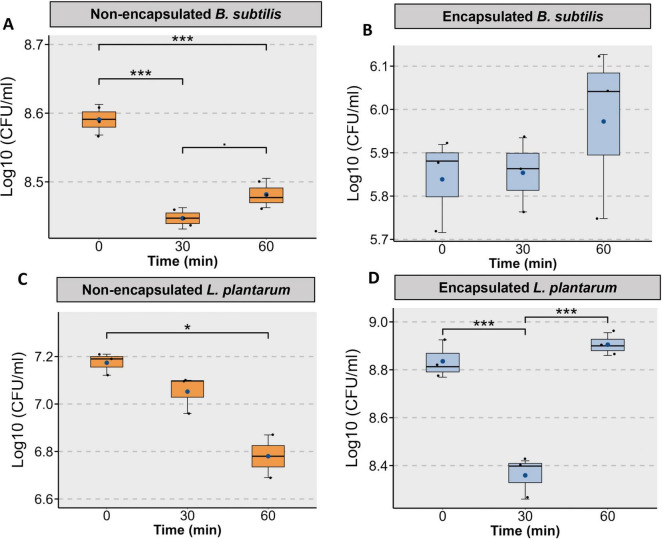
Protective effect of yeast cell wall as an encapsulating agent for *B. subtilis* and *L. plantarum* against damage by simulated gastric juice exposure, *in vitro*. **(A)** Non-encapsulated *B. subtilis* shows a significant decline in viability over time. **(B)** Encapsulated *B. subtilis* exhibits improved survival with less reduction in CFU over the 60-min exposure. **(C)** Non-encapsulated *L. plantarum* shows a notable decrease in viability, particularly after 60 min. **(D)** Encapsulated *L. plantarum* demonstrates a significant decline in viability at 30 min followed by a recovery at 60 min compared to non-encapsulated counterparts (*n* = 3, *p < 0.05, ***p < 0.001).

The viability of non-encapsulated *B. subtilis* was significantly affected during exposure to simulated gastric juice. At 0 min, the bacterial load was approximately 8.59 ± 0.12 Log_10_ CFU/ml. A sharp decline in viability was observed after 30 min of exposure, with CFU counts dropping to around 8.44 ± 0.008 Log_10_ CFU/ml (*p* < 0.001), indicating substantial acid-induced stress (Figure 7A). By 60 min, a slight recovery was noted with CFU levels increasing to 8.48 ± 0.012 Log_10_ CFU/ml (*p* < 0.1), though still significantly (*p* < 0.001) lower than the initial value (0 min). Encapsulated *B. subtilis* exhibited stable viability during exposure to simulated gastric juice over a 60-min period (Figure 7B). The initial count was approximately 5.83 ± 0.06 Log_10_ CFU/ml. Unlike the non-encapsulated form, no decline was observed with time (5.85 ± 0.04 and 5.97 ± 0.11 at 30 and 60 min, respectively).

The viability of *L. plantarum* in simulated gastric juice was significantly affected by encapsulation status. In the non-encapsulated group, the bacterial count decreased steadily over time. The initial CFU level of 7.1 ± 0.02 (Log_10_ CFU/ml) dropped to 6.70 ± 0.09 (Log_10_ CFU/ml) at 60 min, showing a significant reduction (*p* < 0.05) (Figure 7C), indicating susceptibility to acidic conditions. In contrast, encapsulated *L. plantarum* there was a significant initial drop in CFU from Log_10_ 8.8 ± 0.04 to 8.35 ± 0.05 (Log_10_ CFU/ml) at 30 min (*p* < 0.001); however, the count recovered by 60 min, reaching nearly 8.9 ± 0.02 (Log_10_ CFU/ml) again that was not statistically significantly different from the count at 0 min (*p* = 0.29) (Figure 7D).

### 3.7 Viability of *Bacillus subtilis* and *Lactobacillus plantarum* after encapsulation in simulated intestinal fluids

**FIGURE 8:**
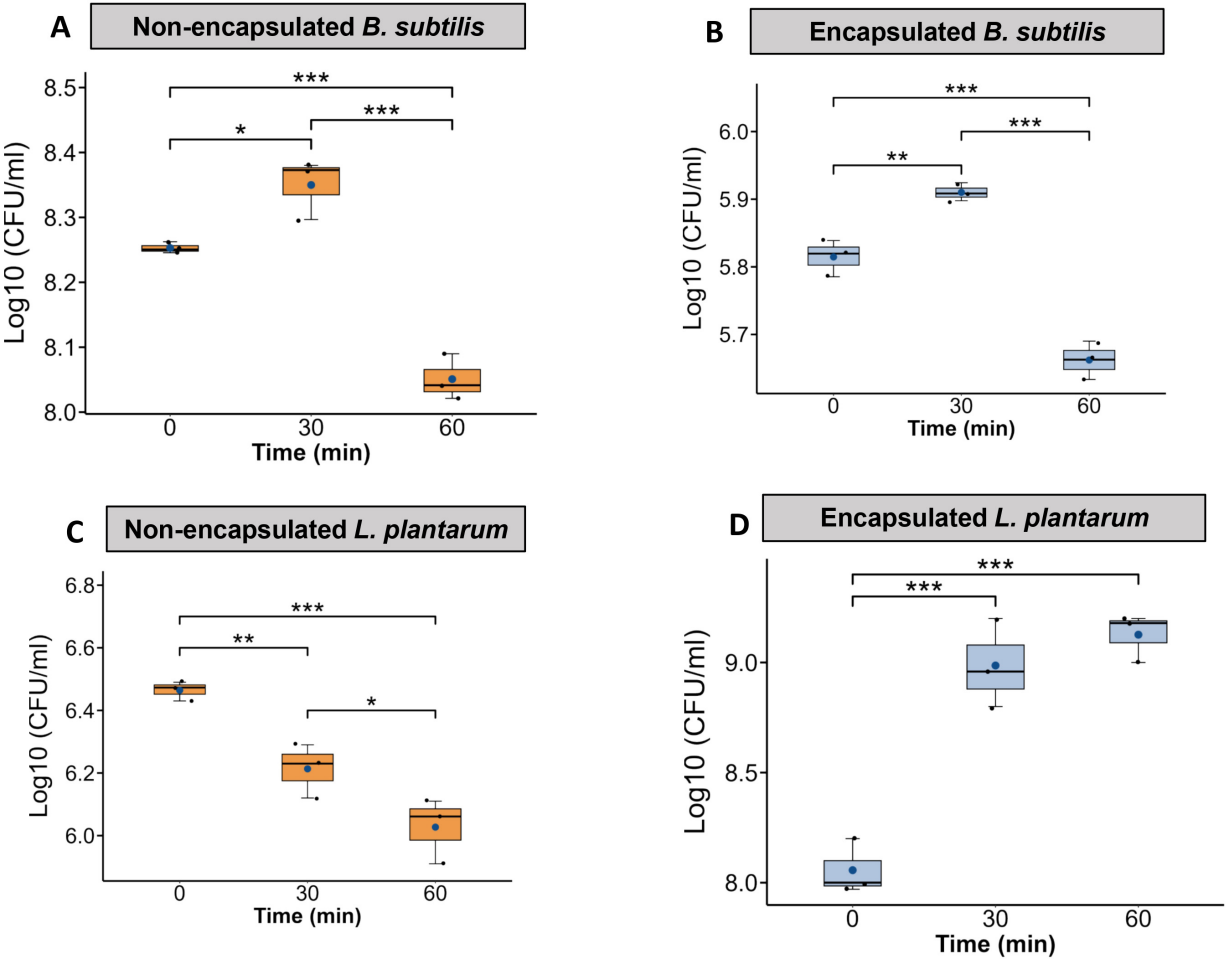
Protective effect of yeast cell wall as an encapsulating agent for *B. subtilis* and *L. plantarum* against damage by simulated intestinal digestive juice exposure, *in vitro*. **(A,B)** Both non-encapsulated and encapsulated forms of *B. subtilis* follow similar trend of reduction in CFU over the 60-min exposure. **(C)** Non-encapsulated *L. plantarum* shows a notable decrease in viability, particularly after 60 min. **(D)** Encapsulated *L. plantarum* demonstrates enhanced resistance to acidic conditions at both 30 and 60 min compared to non-encapsulated counterparts (*n* = 3, ***p < 0.001, **p < 0.01, and *p < 0.05).

The viability of *B. subtilis* and *L. plantarum* in simulated intestinal fluid differed markedly between encapsulated and non-encapsulated forms. Non-encapsulated *B. subtilis* showed an increase in CFU at 30 min (from 8.25 ± 0.005 to 8.34 ± 0.02 Log_10_ CFU/ml) but declined significantly to 8.05 ± 0.02 (*p* < 0.001) at 60 min, indicating reduced stability over time (Figure 8A). A similar trend was observed for encapsulated *B. subtilis* which showed an increased from 5.81 ± 0.015 to 5.91 ± 0.007 (Log_10_ CFU/ml) at 30 min, followed by a decrease to 5.66 ± 0.01 at 60 min (Figure 8B).

Non-encapsulated *L. plantarum* showed a modest and significant decrease at 30 min (6.46 ± 0.01), followed by a drop to 6.02 ± 0.06 at 60 min. These values were significantly lower than the initial viability value of 6.46 ± 0.01 (Log_10_ CFU/ml) (Figure 8C). However, encapsulated *L. plantarum* demonstrated significantly improved survival, with viability rising steadily from 8.05 ± 0.07 (Log_10_ CFU/ml) at 0 min to 8.98 ± 0.11 at 30 min and reaching 9.12 ± 0.06 at 60 min (Figure 8D).

## 4 Discussion

With the rapid expansion of aquaculture over recent decades, considerable attention has been directed toward the application of microbial products as functional feed additives. These supplements have been shown to enhance metabolic performance and disease resistance in aquatic animals (Gora et al., 2019, 2023b), mitigate adverse effects associated with novel feeding strategies (Rehman et al., 2021), and, in some cases, serve as alternative feed ingredients contributing direct nutritional value (Gora et al., 2025). Among these, probiotics have emerged as one of the most promising approaches for improving gut health and reducing reliance on antibiotics. However, their efficacy largely depends on their ability to survive gastrointestinal conditions, adhere to mucosal surfaces, successfully colonize the host, and subsequently exert immunomodulatory and protective effects (Javanshir et al., 2021). In particular, probiotics that improve digestive function and stimulate the gut-associated lymphoid tissue encounter an additional challenge: exposure to harsh pH and possible enzymatic breakdown in the alimentary canal (Mbye et al., 2020). Such conditions can lower the survival and effectiveness of the probiotics. To address this issue, the encapsulation of probiotics has been proposed as a potential eco-friendly approach (Vijayaram et al., 2024). Microencapsulation involves coating the probiotic in a protective membrane that can protect against damage associated with low pH and exposure to enzymes. Several synthetic biodegradable compounds, such as polyvinyl alcohol, polymethacrylates, and poly lactic-co-glycolic acid, have already been explored as encapsulating agents (Barbosa-Nuñez et al., 2025). Though synthetic polymers are excellent in withstanding undesirable environmental conditions in the gut, they are often comparatively more costly and not always “generally regarded as safe” (Satchanska et al., 2024).

Previous studies have employed yeast-derived materials, including purified β-glucans, mannoproteins or chemically modified yeast components, to enhance probiotic stability, adhesion, and functionality (Marchi et al., 2024; Inokuma et al., 2021; Ismail et al., 2025). While these derivatives exhibit notable bioactivities, their extraction involves intensive and costly enzymatic treatments limiting their role primarily to prebiotic or functional additives rather than physical encapsulation matrices (Takalloo et al., 2020; Ismail et al., 2024). Natural encapsulating agents such as pectin, alginate, and chitosan are less costly to develop and require fewer regulatory clearances for use (Supplementary Table 1). Therefore, alginate, pectin, and chitosan are commonly used probiotic encapsulants but exhibit limitations such as acid-induced instability, pH- and enzyme-sensitive degradation, and charge-dependent effects on cell viability and effectiveness varies with molecular characteristics (Lin et al., 2024; Cheng et al., 2025). In contrast, we utilized the yeast cell wall structures derived from *S. cerevisiae* as a natural microencapsulation system. By preserving the multilayered organization of the Baker’s yeast cell wall composed mainly of β-glucans, mannoproteins, and chitin, this approach enables the cell wall to function as a physical protective barrier for probiotic cells. β-glucans and chitin confer rigidity and structural stability to the yeast cell wall and resistance to environmental stressors such as acidic pH, bile salts, and dehydration, whereas mannoproteins, predominantly located in the outer layer, govern surface adhesion, enhancing probiotic retention and stability (Günal-Köroğlu et al., 2024, Ismail et al., 2025). Thus yeast cell wall encapsulation provides a naturally robust, multilayered matrix, offering superior mechanical stability and gastrointestinal resistance and impart biofunctional benefits, including mucoadhesion and immunomodulation, thereby enhancing probiotic survivability and practical applicability (Ballet et al., 2023; Doolam et al., 2025).

In our study, we developed an encapsulating matrix using commercial food-grade baker’s yeast. We evaluated its ability to encapsulate two gut probiotics, protecting them from digestive damage in simulated *in vitro* environments. Yeast cell wall biopolymers consist mostly of β-glucans, mannoproteins, and chitin that can have promising potential as encapsulating materials for probiotics (Utama et al., 2023). Sequential exposure to varying pH solutions washes away the cellular contents of the yeast cells, leaving behind a three-dimensional, hollow, and porous yeast cell wall that facilitates the physical attachment of probiotic bacteria. The protective barrier thus created between the probiotic and the external environment may enhance bacterial viability in the gut and during product manufacture (Van Duc and Dao, 2016; Utama et al., 2023). For efficient attachment, it is essential to develop a mesh matrix that matches the size of the payload microbes. The *L. plantarum* is 0.9–1.2 μm wide and 3–8 μm long, whereas *B. subtilis* is 2–6 μm long and just less than 1 μm in diameter (Errington and Van Der Aart, 2020; Landete et al., 2021). The SEM analysis of the *S. cerevisiae* cell wall exhibited a honeycomb-like configuration, indicating a highly porous architecture (Shen et al., 2015; Shah et al., 2016) conducive to encapsulation processes. The pore size varied between 3 and 6 μm, presenting an optimal pore size range for attachment that is neither too small nor too large for both species of microbes (Saloň et al., 2016; Ren et al., 2018). Moreover, the particle size distribution profiles of the yeast cell wall structure developed in our study had a narrow distribution pattern. A narrow particle distribution pattern indicates a more homogeneous matrix, which is suitable for encapsulation and predictable release behavior (Lalarukh Hussain et al., 2025). Furthermore, the mesh-like porous cell wall structure of baker’s yeast cell wall is composed majorly of β-glucan and mannoprotein as the major components (Utama et al., 2023). Both components have been widely regarded as immunostimulants, as they exhibit the ability to stimulate the activity of specific cells within the gut-associated immune system (Rehman et al., 2022b). β-glucan from microbial sources can also modulate the expression of gene connected to lipid metabolism and antioxidant status (Gora et al., 2022, 2023a) and inflammatory response (Rehman et al., 2021, 2023) in fish. Therefore, the use of the baker’s yeast cell wall as an encapsulating agent performs the dual function of helping the probiotic establish itself in the gut while at the same time providing an immunostimulatory effect on the host mucosal tissue.

The SEM showed *B. subtilis* and *L. plantarum* randomly distributed within the yeast-derived matrix. However, there was a distinct morphological characteristic revealed by DLS, showing an increased particle size of 4.9 μm *B. subtilis* and 3 μm for *L. plantarum*. The increase in the size of the yeast cell wall occurs when the encapsulating material absorbs water or interacts with the bacteria, likely leading to a broader size distribution –a phenomenon that has also been observed in *B. subtilis* encapsulated in polyvinyl alcohol (David et al., 2021). The discrepancy between particle sizes obtained by DLS and SEM arises from their distinct measurement principles. DLS measures the hydrodynamic diameter of yeast cell wall–based microcapsules in aqueous suspension, incorporating hydration layers and matrix swelling, which results in larger apparent sizes (Bhattacharjee, 2016). In contrast, SEM determines the dry physical diameter under vacuum, where dehydration and partial structural collapse may occur. Moreover, DLS provides intensity-weighted size distributions that are highly sensitive to larger particles or minor aggregation, whereas SEM yields number-based measurements (Tarrés et al., 2022).

Interaction with water and bacteria also leads to changes in the electrostatic and biochemical profile of the complex (Baek et al., 2024), which likely induces clumping and aggregation of the bacterial cells. It has been claimed that such interactions can contribute to enhanced structural integrity and may further improve the stability of encapsulated probiotics during storage (Utama et al., 2023). In addition, *L. plantarum* encapsulation resulted in a span of 1.6, indicative of moderate polydispersity. Encapsulation of *L. plantarum* using whey protein and maltodextrin also produced larger and more polydisperse particles, which may be attributed to aggregation during drying and variable interaction with wall materials (Rajam et al., 2012).

Infrared spectroscopy provides molecular-level insights, enabling the identification of surface functional groups (Balan et al., 2019). FTIR spectral analysis provided evidence supporting the successful microencapsulation of *B. subtilis* and *L. plantarum* within the yeast cell wall matrix. Encapsulation of *B. subtilis* and *L. plantarum* within the yeast cell wall is governed by non-covalent interactions between functional groups on the probiotic surface and the yeast matrix. The primary contributors are hydroxyl (–OH) groups of yeast β-glucans and mannans, as evidenced by broad and attenuated O–H stretching bands (∼3400–3500 cm^–1^), reflecting extensive hydrogen bonding with bacterial surface polysaccharides and proteins (Ismail et al., 2025). Attenuation and partial overlap of amide I and II bands (∼1650 and ∼1540 cm^–1^) further indicate close association of *L. plantarum* surface proteins with yeast polysaccharides. In *B. subtilis*, the involvement of phosphate-containing groups (1229–1150 cm^–1^), linked to teichoic acids, suggests an additional electrostatic interaction, accounting for more pronounced spectral changes than in *L. plantarum*. Enhanced glycosidic and ether vibrations (C–O–C, C–O; 1200–900 cm^–1^) confirm dominance of the yeast polysaccharide matrix. The absence of new absorption bands demonstrates that encapsulation occurs via hydrogen bonding, electrostatic attraction, and physical adsorption, preserving probiotic structural integrity (Ahmadi et al., 2025).

The colloidal stability of the microparticles is of critical importance to assess the shelf-life and potential for successful probiotic delivery (Ayala et al., 2013). Zeta potential analysis is a reliable method for confirming the colloidal stability of microparticles. The native yeast cell wall, without the probiotic payload, had an overall negative surface charge (−15.7 mV), which is considered sufficient to generate electrostatic repulsion, prevent particle aggregation, and maintain colloidal stability (Lin et al., 2021; Ashraf et al., 2021). Strikingly, the yeast microcapsules loaded with *B. subtilis* and *L. plantarum* exhibited even more negative zeta potentials at −32.73 ± 1.39 and −30.36 ± 0.42 mV, respectively, likely indicating a greatly enhanced colloidal stability due to stronger electrostatic repulsion (Nejadmansouri et al., 2023). However, while higher absolute zeta potential values (≤−30 mV) suggest improved electrostatic stabilization, colloidal stability is also influenced by surface hydrophobicity and polymeric surface composition introduced during encapsulation. In yeast cell wall-encapsulated probiotics, both electrostatic repulsion and altered surface chemistry (β-glucan and mannoprotein exposure) likely act synergistically to reduce aggregation, rather than zeta potential alone. The observed difference in zeta potential between *L. plantarum*– and *B. subtilis*–encapsulated microcapsules can be attributed to strain-specific cell surface chemistry and its interaction with the yeast cell wall matrix. *L. plantarum* (Gram-positive lactic acid bacterium) possesses a cell envelope rich in teichoic and lipoteichoic acids, which can enhance electrostatic interactions with components of the yeast cell wall, such as β-glucans and mannoproteins. In contrast, *B. subtilis*, although also Gram-positive, has a distinct cell wall architecture and surface protein composition, including spore-associated structures and different polysaccharide profiles, which modulate its net surface charge differently upon coating (Nejadmansouri et al., 2023). As a result, the final surface charge of the microcapsules reflects a combined contribution of the yeast cell wall matrix and the intrinsic surface properties of each probiotic strain, leading to the observed variation. *L. plantarum* has been shown to induce a stable colloidal structure in cellulose–chitosan hybrid materials with a zeta potential of −26 mV, *E. coli* encapsulated with yeast membranes exhibited −13.1 mV, and *B. subtilis* encapsulated with oat β-glucan reached −21.6 mV from −19.0 in unloaded microcapsules (Lin et al., 2021; Afzaal et al., 2022; do Carmo Alves et al., 2023). Biologically, negatively charged particles generally exhibit improved dispersion uniformity and reduced nonspecific protein adsorption, leading to lower cytotoxicity and slower opsonization compared with highly cationic particles (Bhattacharjee, 2016; Li et al., 2022). Although electrostatic repulsion may limit nonspecific membrane adhesion, effective cellular interactions can still occur via charge-independent mechanisms such as receptor-mediated uptake and pattern-recognition pathways, particularly relevant for immune modulation applications (Verma and Stellacci, 2010). Collectively, our finding supports the potential of yeast cell wall-based microencapsulation as an effective and stable strategy for probiotic delivery.

After confirming the stability of the resulting matrix through microencapsulation of probiotics into yeast cell walls, we evaluated the encapsulation efficiency of the two probiotic strains. Encapsulation efficiency is a crucial criterion for understanding the efficiency of microencapsulation processing and ruling out significant compromises to the viability of probiotics during the development of a microencapsulated matrix. The encapsulation process involves exposing viable bacteria to several harsh physicochemical conditions, including exposure to detergent, homogenization, and lyophilization, which can hamper the viability of the microbes. The bacterial growth curve analysis was performed to identify the exponential phase of growth, which was used as the reference point for harvesting cells to ensure accurate, reproducible, and physiologically comparable cell dosages across all treatments. Cells collected at the same phase of culture exhibit more uniform metabolic activity, membrane integrity, and stress tolerance, thereby minimizing variability in subsequent assays such as encapsulation efficiency, simulated gastrointestinal survival, and viability assessment. CFU values derived from the growth curves were used to standardize the initial inoculum concentration prior to encapsulation and exposure experiments, ensuring that observed differences arose from treatment effects rather than growth-stage–dependent variability. To rule out any significant loss of viability by microencapsulation, a high initial count was maintained (∼Log_10_ 9–10 CFU/mL) for both strains, consistent with optimal probiotic culture conditions prior to processing (Mokhtari et al., 2017). In this experiment, a parallel group (non-encapsulated) was maintained to reveal the effects of harsh physicochemical processes without the encapsulating material. Previous studies have demonstrated improved probiotic viability and improved encapsulation of bioactive compounds using yeast cell-based materials (Van Duc and Dao, 2016; Dadkhodazade et al., 2021). In our study, though a marked reduction in CFU was observed post-encapsulation, the final viability was significantly higher compared to the non-encapsulated counterparts. The lower viable count of the non-encapsulated *B. subtilis* and *L. plantarum* indicates the effectiveness of the encapsulating material against the encapsulation-induced physicochemical fluctuations. The observed reduction in CFU following encapsulation is consistent with previous reports indicating that microencapsulation can impose transient physiological stress on probiotic cells during processing, leading to a modest initial loss in culturability (Tülek et al., 2025). Despite this initial decline, both *B. subtilis* and *L. plantarum* exhibited significantly higher survival in the encapsulated form compared with their non-encapsulated counterparts, highlighting the protective role of the yeast cell wall matrix. A previous study obtained similar results with an efficiency of 88% for the *L. acidophilus* and *B. bifidum* after encapsulation in yeast cell walls (Mokhtari et al., 2017). These results indicate that yeast cell wall-based microencapsulation offers substantial protection to probiotic cells, likely by forming a physical barrier that reduces cell injury during homogenization, freeze-drying, and detergent exposure. Although both strains benefited from encapsulation, *B. subtilis* showed marginally higher survival compared to *L. plantarum*. This difference could be attributed to the intrinsic robustness of *B. subtilis*, a spore-forming bacterium known for its higher resistance to stress factors (Gautério et al., 2023). The enhanced structural integrity and storage stability of the encapsulated probiotics arise from electrostatic interactions, hydrogen bonding, and hydrophobic interactions between probiotic cell surfaces and yeast cell wall components such as β-glucans and mannoproteins. These interactions promote strong coating cohesion, generate a highly negative zeta potential that reduces aggregation, and forms a semi-permeable barrier limiting moisture and oxygen diffusion during storage. Collectively, this stabilizing network preserves cell membrane integrity and improves long-term probiotic viability (Lim et al., 2025; Cardoso-Cardenas et al., 2025).

Next, we aimed to evaluate whether the encapsulation process offers any tangible benefits to the viability of probiotics against digestive enzymes *in vitro* conditions. Yeast cell wall encapsulation enhanced the survivability of *B. subtilis* and *L. plantarum* under simulated gastric conditions. Non-encapsulated *B. subtilis* exhibited a significant decline in viability within the first 30 min of exposure, underscoring its susceptibility to acidic stress. Although a slight recovery was observed at 60 min, the overall viability remained compromised. In contrast, the encapsulated form maintained stable CFU levels throughout the exposure period, indicating that yeast cell wall encapsulation effectively mitigated acid-induced stress and preserved bacterial viability. Previous studies have confirmed that yeast cell walls that are mainly composed of β-glucans and mannoproteins can protect against acidic environments (Shah et al., 2016; Mokhtari et al., 2017). Similarly, *L. plantarum* showed improved stability when encapsulated, whereas the non-encapsulated form displayed a steady decline in viability over time. The encapsulated group initially experienced a reduction in CFU count, likely due to partial acid penetration or a transient stress response. However, the subsequent recovery to initial levels within 60 min suggests that encapsulation not only provided protection but may have also facilitated resilience or regrowth under harsh conditions. This apparent increase in CFU for encapsulated *L. plantarum* and *B. subtilis* is also likely due to time-dependent protection and gradual release of viable cells from the encapsulation matrix, together with recovery of sublethally injured cells during enumeration, rather than active growth under acidic conditions. This phenomenon has been previously reported (Han et al., 2024).

For the intestinal fluid exposure, there was a more or less similar trend observed for non-encapsulated and encapsulated *B. subtilis*, i.e., a significant increase in CFU at 30 min, followed by a decline at 60 min, suggesting a limited protective effect of yeast cell wall encapsulation against intestinal enzyme-induced damage for this strain. The reduction in viable counts is likely due to the breakdown of their yeast cell wall (Gautério et al., 2023). In contrast, *L. plantarum* demonstrated significant susceptibility to incubation in simulated intestinal fluid but improved survival when encapsulated, with CFU counts rising steadily over the 60 min. The increase in viable counts after 60 min of exposure to simulated intestinal fluid likely reflects physiological recovery of stressed cells and time-dependent release from the encapsulation matrix under near-neutral conditions, allowing more cells to regain viability and be enumerated as CFU (Rojas-Espina et al., 2024). This observation is consistent with previous reports showing that encapsulation enhances probiotic survival in simulated gastrointestinal environments compared with free cells, particularly when yeast-derived materials are used as protective matrices (Shah et al., 2016; Mokhtari et al., 2017). The stronger protection observed for encapsulated *L. plantarum* compared with *B. subtilis* can be attributed to species-specific differences in gastrointestinal adaptation and survival strategies (Golnari et al., 2024). *L. plantarum*, a gut-adapted lactic acid bacterium with inherent bile tolerance and adhesion capacity, may interact more effectively with the encapsulation matrix, promoting improved intestinal survival (Shoukat et al., 2025). In contrast, *B. subtilis* is primarily delivered as spores, which rely on dormancy for resistance rather than sustained protection of vegetative cells, potentially limiting matrix-mediated viability enhancement in intestinal conditions (Williams and Weir, 2024; Ren et al., 2025). This highlights the microbe-specific nature of encapsulation efficacy that should be considered in probiotic product design.

While the present study demonstrates promising protective effects of the yeast cell wall–based encapsulation system under controlled *in vitro* conditions, several limitations should be acknowledged. The findings are based solely on *in vitro* evaluations, and *in vivo* studies are necessary to confirm probiotic survival and functional efficacy under practical conditions. Storage stability was assessed under limited environmental parameters, which may not fully reflect commercial handling and feed-processing scenarios. Finally, the scalability and feasibility of the encapsulation process require further economic analyses and validation before industrial application.

## 5 Conclusion

This study demonstrates that baker’s yeast cell wall–based microencapsulation is an effective, rapid, and industry-feasible strategy for enhancing probiotic stability and gastrointestinal tolerance. The hollow, porous yeast cell wall matrix provided substantial protection to encapsulated probiotics against acid and digestive enzyme-induced stress under simulated gastrointestinal conditions, resulting in significantly improved viability compared with non-encapsulated cells. Importantly, the protective efficacy was strain dependent, reflecting differences in probiotic surface chemistry and their physicochemical interactions with yeast cell wall components. Overall, these findings support the suitability of yeast cell wall microencapsulation as a safe and biodegradable platform for oral probiotic delivery in functional feed and pharmaceutical applications. Future studies should focus on *in vivo* validation of this system in teleostean and mammalian models to confirm its translational relevance and host-specific performance.

## Data Availability

The original contributions presented in this study are included in this article/Supplementary material, further inquiries can be directed to the corresponding author.
