# Supplementary material for: Microencapsulation of Lactobacillus plantarum and Bacillus subtilis using baker’s yeast cell wall: characterization and stability assessment under stress conditions
Source: Front Microbiol. 2026 Apr 1;17:1719665. doi: 10.3389/fmicb.2026.1719665 (PMC13079046; doi:10.3389/fmicb.2026.1719665)
Supplement: Supplementary file 1 [file Table_1.docx]

| Encapsulation material / system | Probiotic strain(s) | Encapsulation method | Stress conditions evaluated | Key outcomes on probiotic stability | Reference |
| --- | --- | --- | --- | --- | --- |
| Alginate beads | *Lactiplantibacillus plantarum* | Emulsification | Simulated GI (acid, bile), storage | Good protection in gastric simulation; improved shelf-life when combined with co-polymers | Bautista Villarreal et al., 2023. |
| Alginate–chitosan (layered) | *L. fermentum* | Ionic-gelation | Simulated GI, freeze-drying | Enhanced acid and bile tolerance vs alginate alone; improved survival after drying | Senthil Kumar et al., 2025 (chitosan–alginate synbiotic capsules |
| Alginate–pectin blend | *L. bulgaricus* | Extrusion | Simulated gastric & intestinal fluids | Better acid protection and textural strength vs single polymer beads | Hu et al., 2021 (alginate/pectin hydrogel microspheres). |
| Chitosan nanoparticles (CNPs) | *Lactobacillus plantarum* | Ionic-gelation | Thermal, oxidative stress; application trials | Improved thermal tolerance and functional delivery in some formulations | EI Hassanen et al., 2023 |
| Spray-dried with skim milk and yeast extract | *Lactobacillus plantarum* | Spray drying | Heat (spray), storage stability | Good shelf stability when optimized; survival strongly dependent on inlet/outlet temps and protectants | Kaymak Ertekin et al., 2024 (spray-drying optimisation for L. plantarum).) |
| Baker’s yeast cell wall (YCW) — present study | *Lactobacillus plantarum* & *Bacillus subtilis* | Physical attachmentt / adsorption into *S. cerevisiae* cell wall matrix | Simulated GI (acid, bile), Freeze drying | Significantly enhanced stress tolerance and structural stability compared with unencapsulated cells | Present study |

**Microencapsulation of *Lactobacillus plantarum* and *Bacillus subtilis* using baker's yeast cell wall: characterization and stability assessment under stress conditions**

**Supplementary Table 1. Comparative summary of previously published studies relevant to the present work**

**References:**

Bautista Villarreal, M., Castillo Hernández, S. L., Lopez Uriarte, S., & Barron Gonzalez, M. P. (2023). Encapsulation of Lactiplantibacillus plantarum and beetroot extract with alginate and effect of capsules on rheological properties and stability of an oil-in-water emulsion model food. Polish Journal of Food and Nutrition Sciences, 73(3), 242-252.

Senthil Kumar, S., & Sheik Mohideen, S. (2025). Encapsulation of L. fermentum with chitosan-alginate enhances its bioactivity against acrylamide toxicity in D. mel. Scientific Reports, 15(1), 11324.

Hu, X., Liu, C., Zhang, H., Hossen, M. A., Sameen, D. E., Dai, J., ... & Li, S. (2021). In vitro digestion of sodium alginate/pectin co-encapsulated Lactobacillus bulgaricus and its application in yogurt bilayer beads. International Journal of Biological Macromolecules, 193, 1050-1058.

Hassanen, E. I., Ahmed, L. I., Fahim, K. M., Shehata, M. G., & Badr, A. N. (2023). Chitosan nanoparticle encapsulation increased the prophylactic efficacy of Lactobacillus plantarum RM1 against AFM1-induced hepatorenal toxicity in rats. Environmental Science and Pollution Research, 30(59), 123925-123938.

Kaymak Ertekin, F., Köprüalan Aydın, Ö., & Altay, Ö. (2024). Enhancing viability of Lactobacillus plantarum BG24 through optimized spray drying: insights into process parameters, carrier agents, comparative analysis with freeze drying, and storage condition influences. Food Science & Nutrition, 12(12), 10330-10346.

**Supplementary Picture:** Representative SEM images of probiotics at different magnifications
